# Personalised estimation of exposure to ambient air pollution and application in a longitudinal cohort analysis of cognitive function in London-dwelling older adults

**DOI:** 10.1038/s41370-025-00745-7

**Published:** 2025-01-14

**Authors:** Dylan Wood, Dimitris Evangelopoulos, Nutthida Kitwiroon, Gregor Stewart, Tuan Vu, James Smith, Sean Beevers, Klea Katsouyanni

**Affiliations:** 1 Environmental Research Group, School of Public Health, Imperial College London, London, UK; 2 MRC Centre for Environment and Health, Environmental Research Group, Imperial College London, London, UK; 3 NIHR HPRU in Environmental Exposures and Health, Imperial College London, London, UK; 4 Guy Carpenter, London, UK; 5 Department of Hygiene, Epidemiology and Medical Statistics, School of Medicine, National and Kapodistrian University of Athens, Athens, Greece

**Keywords:** Air pollution, Personal exposure, Epidemiology, Exposure assessment, Exposure modeling

## Abstract

**Background:**

Accurate estimates of personal exposure to ambient air pollution are difficult to obtain and epidemiological studies generally rely on residence-based estimates, averaged spatially and temporally, derived from monitoring networks or models. Few epidemiological studies have compared the associated health effects of personal exposure and residence-based estimates.

**Objective:**

To evaluate the association between exposure to air pollution and cognitive function using exposure estimates taking mobility and location into account.

**Methods:**

Residence-based dispersion model estimates of ambient NO_2_, PM_10_ and PM_2.5_ were assigned to 768 London-dwelling participants of the English Longitudinal Study of Ageing. The London Hybrid Exposure Model was implemented to adjust estimates per pollutant to reflect the estimated time-activity patterns of each participant based on age and residential location. Single pollutant linear mixed-effects models were fit for both exposure assessment methods to investigate the associations between assigned pollutant concentrations and cognitive function over a follow-up period of up to 15 years.

**Results:**

Increased long-term exposures to residence-based ambient NO_2_ (IQR: 11.10 µg/m^3^), PM_10_ (2.35 µg/m^3^), and PM_2.5_ (2.50 µg/m^3^) were associated with decreases of −0.10 [95% CI: −0.20, 0.00], −0.07 [−0.11, −0.02] and −0.14 [−0.21, −0.06], respectively, in composite memory score. Similar decreases were observed for executive function scores (−0.38 [−0.58, −0.18], −0.11 [−0.20, −0.02] and −0.14 [−0.29, 0.01], respectively). When applying personalised exposure estimates, which were substantially lower, similar decreases were observed for composite memory score per IQR, but a consistent pattern of slightly more adverse effects with executive function score was evident.

**Impact Statement:**

The present study constructed a framework through which time-activity information derived from a representative sample could be applied to estimates of ambient air pollution concentrations assigned to individuals in epidemiological cohort studies, with the intention of adjusting commonly used residence-based estimates to reflect population mobility and time spent in various microenvironments. Estimates of exposure were markedly lower when incorporating time-activity, likely because people in European populations spend a large proportion of their time indoors, where their exposure to ambient air pollution may be reduced through infiltration, which is not taken into account in residence-based ambient estimates. Further work into such methods could provide insights into the efficacy of personalising exposure estimates.

## Introduction

A large number of epidemiological studies to date have provided evidence of adverse relationships between exposure to ambient air pollutants and mortality or morbidity [1, 2], including a growing body of evidence in recent years suggesting associations between increased exposure to several pollutants and reduced cognitive ability in older adults [3, 4]. However, accurate estimation of individual-level exposure can often be difficult to obtain and static estimates based on the individuals’ residence are most often applied in such studies [5].

Long-term estimates of exposure to ambient pollutants in epidemiological studies are most often derived from fixed-site monitoring networks, usually applying averaged concentrations from the closest monitor to an individual’s residence, or concentrations are modelled at a fine spatial scale (e.g., 20 × 20 m [6]). A potential issue with assigning residence-based estimates is the fact that an individual’s time-activity patterns and mobility through space are not accounted for, which may lead to inaccurate estimates of exposure [7]. Furthermore, averaging residence-based concentrations over a long time period (e.g., one year) as a measure of exposure for an individual, assumes that total personal exposure to ambient concentrations (including exposure whilst indoors and travelling) is equal, or at least highly correlated, to that of the annual concentration either measured at the nearest monitor to the home or modelled at the residence itself. Total personal exposure to ambient concentrations of air pollutants is difficult to measure given the cost involved in personal monitoring campaigns in large enough populations to detect associations, the inherent inconvenience for participants and the inability to separate concentrations from indoor- and outdoor-generated sources within personal monitoring campaigns themselves [8].

Individuals are exposed to air pollutants at varying concentrations whilst indoors, outdoors or travelling in different microenvironments [9]. Studies have shown that people generally spend more than two-thirds of their time indoors [8], although this can vary between populations, with Londoners estimated to spend up to 95% of their time indoors either at home or elsewhere [10]. Exposure to ambient concentrations when indoors is typically lower than when outdoors, with a study across five European cities finding exposure to outdoor-generated PM_2.5_ to be 16–20% lower when indoors [11]. However, infiltration of ambient concentrations to indoor environments varies between building types given the differences in structures of building envelopes and permeability of exposed facades [12, 13]. Exposure when travelling has also been shown to vary between transportation modes. A review of studies assessing exposure to PM_2.5_ across several common transportation modes (car, cycling, diesel/electric bus and walking) by de Nazelle et al. [14] found pedestrians to be consistently less exposed in comparison to other forms of travel. Investigations in London have shown variability in exposure between transportation modes, as well as differences in exposure reported between the London Underground system and those observed at background or roadside monitoring stations (reaching up to 15 times greater concentrations of PM_2.5_ on the London Underground network in comparison to surface levels), suggesting plausible evidence that population mobility and commuter trips may greatly influence an individual’s personal exposure [15, 16].

Few studies have investigated the correlation between measured personal exposure and residence-based estimates and the body of work is still limited. If residence-based estimates are consistently highly correlated with measured personal concentrations, the application of monitor/modelled estimates at the residence may sufficiently capture personal exposure and be applied as accurate proxies in epidemiological studies. A number of studies have shown this to be the case in specific scenarios [17–20], however, more work is required to fully understand the extent to which these high correlations can be generalised across all study settings. Additionally, previous work has compared health effect estimates in epidemiological studies between the application of residence-based estimates and measured personal exposure. In the majority of instances, little to no difference has been observed in effect estimates [20–22], suggesting that the application of residence-based estimates yields the same results, precluding the need for intensive personal monitoring campaigns for epidemiological studies. Further work is also required in this area of research.

The present study aimed to develop a methodology through which indirect adjustment of residence-based estimates of exposure to ambient air pollutants could be performed through the incorporation of external time-activity and population mobility information derived from a representative sample population; providing exposure estimates that may be closer to actual personal exposure to ambient air pollution. The application of this methodology was then applied to the findings of previous work [23], in which adverse associations were observed between long-term residence-based estimates of ambient air pollutants and cognitive test performance in a London-dwelling subset of a national cohort of older adults. Modelled estimates of residential exposure at a fine spatial scale were utilised and annual average concentrations were assigned to 768 London-dwelling participants of the English Longitudinal Study of Ageing (ELSA), investigating the relationship between increasing exposure and performance on cognitive tests over a 15-year follow-up period. The application of the London Hybrid Exposure Model [10] (LHEM) provided an indirect estimate of personal exposure to pollutants from ambient sources, deriving estimated time-activity patterns and population mobility to inform microenvironment modelling of exposure in a representative sample of Londoners (participants of the London Travel Demand Survey; LTDS). Effect estimates of increasing exposure to NO_2_, PM_10_ and PM_2.5_ on repeated measures of cognitive function were compared between both exposure estimation methods.

## Materials and methods

### Study population and health outcome

ELSA is an ongoing interdisciplinary cohort study of adults aged ≥50 years at baseline from across England [24, 25]. ELSA was established in 2002 with biennial follow-up interviews conducted. The baseline cohort (*n* = 11,391) was drawn from households that had responded to the Health Survey for England (HSE) in 1998, 1999 and 2001 and is broadly representative of the English population aged 50 years and older in terms of sociodemographic characteristics [25]. Analysis in the present study included 768 London-dwelling core members (provided by the National Centre for Social Research; NatCen; https://natcen.ac.uk/s/elsa-50-health-and-life) that undertook an in-person baseline interview (2002–2003) and at least one subsequent in-person follow-up (2004–2017). ELSA data was provided by NatCen observing all General Data Protection Regulation (GDPR) procedures and no data were collected under the responsibility of the present study. The data provided was approved by the NatCen data release review panel and remained anonymised throughout the analysis.

Cognitive test scores administered at interviews were used to measure cognitive function. ELSA participants were asked at each interview to undertake a cognitive test battery, including tests of memory and executive function [24]. The present study implemented the same protocol as previous studies [26–28] and calculated a score ranging from 0 to 20 as a composite measure of memory. Executive function was tested using an animal naming test in which the total number of animals named within one minute was used as the executive function score. Both memory and animal naming tests were included in all ELSA follow-up interviews, with the exception of the 2012-13 follow-up interview which did not include the animal naming test. Tests of word recall and the animal naming test have been shown to display good construct validity both in ELSA and other cohorts [29, 30].

### Exposure assessment

Annual average concentration estimates of NO_2_, PM_10_ and PM_2.5_ were assigned to London-dwelling ELSA individuals using a dispersion model [31] (Community Multiscale Air Quality Urban; CMAQ-urban). These estimates were then modified to reflect estimated time-activity patterns based on age and residence in London, derived from a representative sample of the London population (the London Travel Demand Survey; LTDS) and a microenvironment modelling framework [10] (the LHEM), providing two measures of exposure: a residence-based annual average and an indirect measure of personal exposure to pollutants from ambient sources.

#### Residence-based annual average concentration: CMAQ-urban

Annual average residence-based estimates of NO_2_, PM_10_ and PM_2.5_ were estimated at the residential postcode of ELSA participants using the CMAQ-urban dispersion model, derived from hourly concentrations estimated at a 20 × 20 m scale, for the years 2004 and 2012. The residential postcode was made available for linkage for each ELSA participant and CMAQ-urban estimates were, therefore, averaged for each pollutant at the postcode level by calculating the annual average concentration within the grid cell containing the postcode centroid. Each postcode in London is comprised of 18 households on average. Validation of the CMAQ-urban model estimates showed good performance in comparison with a holdout data set of measured concentrations across a national fixed-site monitoring network (Supplementary Material Fig. S1 and Table S1 for the whole of the UK); also described in Wood et al. [23]. Validation of CMAQ-urban modelled concentrations against measured concentrations at a daily level has also been conducted for London [6] and provided cross-validated *R*^2^ values of >0.70 for PM and NO_2_.

For participant interviews conducted between 2002 and 2009 residential concentrations were assigned the 2004 estimates, whilst for interviews conducted between 2010 and 2017 residential concentrations were assigned the 2012 estimates, based on the assumption that spatial variability remains consistent in adjacent years. Moving homes during follow-up was taken into account with ELSA participants that moved home during the course of follow-up assigned concentration estimates applied to the residential postcode at the time of interview.

To protect respondents’ identity and eliminate the possibility of post hoc identification of respondent postcodes through point-estimate combinations, postcode-level annual average pollutant estimates across London were classified into categories (using deciles of the England-wide distribution for each pollutant; Supplementary Material Table S2). A full description of the methodology is provided by Wood et al. [23].

#### Personalised exposure estimation: the London Travel Demand Survey (LTDS) and the London Hybrid Exposure Model (LHEM)

The LTDS is an ongoing annual survey conducted by Transport for London (TfL) aimed at understanding the movement of London’s population throughout the city, with a focus on transport use. The LTDS attempts to maintain a representative sample of Londoners and surveys residents aged five years and older from approximately 8000 households per year [32]. Information collected during surveys includes sociodemographic data such as age, gender, marital status and residential address, as well as socioeconomic information including employment status, occupation and income. Additionally, information on when the individual was at home, indoors elsewhere or travelling is recorded. Data for each trip undertaken during the given survey day (trip origin, destination, start time and mode(s) of transport taken) is also recorded. The present study included LTDS data collected between the years 2005 and 2010 for 19,349 London residents aged 50 years or older (Supplementary Fig. S2).

The LHEM utilises LTDS data in order to create a minute-by-minute reconstruction of each individual’s survey day, before estimating personal exposure based on the microenvironment in which the individual was located at each minute. For each individual, recorded survey data on time spent at home, indoors elsewhere and travel information are used to reconstruct the given survey day utilising trip-route-simulation application programming interfaces (APIs) and start/end coordinates provided for each trip recorded. Trip-route simulation APIs incorporated in LHEM modelling include the TfL Journey Planner for public transport trips, Google Directions for cycling, Project OSRM for road vehicle trip simulations and The Open Route Service for walking trips [10]. Hourly concentrations of NO_2_, PM_10_ and PM_2.5_ estimated by the CMAQ-urban dispersion model [31] at 20 × 20 m resolution for the year 2011 are then assigned to the estimated location of the individual for each minute (estimated via route simulation when travelling or the use of exact coordinates when indoors). Microenvironment models are implemented to modify assigned point-time location concentration estimates. For in-building exposure, indoor/outdoor ratios are modelled utilising the London housing stock to estimate building type where applicable [33]. In-vehicle exposure is calculated through the mass balance equation, incorporating vehicle volume (estimated using recorded vehicle type), surface area and air exchange rates. When travelling on the London Underground, measurement data were used to assign concentration estimates [10]. When LTDS respondents reported as walking, cycling or being a rider/passenger of a motorcycle, ambient CMAQ-urban concentration estimates were assigned based on point-time location. Additionally, hourly residential CMAQ-urban estimates were assigned at the home address of each individual throughout the given survey day.

#### Calculation of personal exposure to pollutants from ambient sources for ELSA participants

The reconstructed survey day for each individual at one-minute resolution was used to calculate a personal exposure factor for each pollutant (the ratio at each minute between the residential CMAQ-urban concentration and the point-time CMAQ-urban estimate adjusted via the LHEM according to microenvironment; Eq. 1). CMAQ-urban 20 × 20 m estimates covering London for the year 2011 were included in the LHEM. The mean of these minute-by-minute factors was taken to calculate an average difference between residential concentrations and personal time-activity reflective concentrations for the given survey day for NO_2_, PM_10_ and PM_2.5_ per individual. The daily mean factors for each pollutant were then aggregated both spatially and by age. The mean factor for two age groups (50–64 and 65 years and older) per postcode sector in London (1445 postcode sectors within the city; 220 postcodes per postcode sector on average; Supplementary Table S4) was calculated. The amount of time spent in each microenvironment was also calculated at the individual level and aggregated by age group and at the same spatial scale. Microenvironments were grouped into indoors at home, indoors out of the home, walk/cycle/motorcycle, London Underground/Docklands Light Railway, train and road vehicle. Descriptive statistics were produced to understand the differences in travel behaviours and time spent indoors between age groups. For each participant of the ELSA cohort, the average factors corresponding to their age group and residential postcode sector were applied and their residential concentration estimate was transformed into a personalised exposure assuming the time-activity patterns to be similar to the LTDS participants of the same age group and residential area.

1 $$\begin{array}{c}{{{\boldsymbol{Ratio}}}}_{{{\boldsymbol{i}}}}={{{\rm{LHEMAdjustedConc}}}}_{{{\rm{i}}}}/{{{\rm{ResidentialPollutantConc}}}}_{{{\rm{i}}}},{{\rm{Individuals}}}\; {{\rm{i}}}=1\,\ldots ,19,349\\ {{{\boldsymbol{AF}}}}_{{{\boldsymbol{jk}}}}={{{\boldsymbol{Ratio}}}}_{{{\boldsymbol{i}}}},{{\rm{for}}}\; {{\rm{each}}}\; {{\rm{postcode}}}\; {{\rm{sector}}}\; {{\rm{j}}}=1,\,\ldots ,\,1,445,{{\rm{Age}}}\; {{\rm{group}}}\; {{\rm{k}}}=1,\,\ldots ,\,2\end{array}$$

Calculation of the ratio between hourly residential (CMAQ-urban modelled) and LHEM-adjusted (reflective of location and microenvironment) concentrations assigned at each minute for each LTDS individual aged 50 years and older (i) for NO_2_, PM_10_ and PM_2.5_. Ratios were aggregated per individual and then by age group (k) and postcode sector (j) to provide adjustment factors (AF).

### Statistical analysis

The present study investigated associations between CMAQ-urban residence-based estimates of NO_2_, PM_10_ and PM_2.5_ concentrations, as well as personalised estimates, and repeated measures of cognitive function in the ELSA cohort. Single pollutant linear mixed-effects models were constructed with the cognitive test score (as a continuous variable) at each follow-up included as the dependent variable. Repeated measurements (cognitive testing at each follow-up interview) for each ELSA participant were accounted for via the inclusion of a random intercept per individual. Concentration estimates for both metrics were assigned as the exposure variable to the previous interview (or baseline for the baseline interview) in order to assess the long-term effects of pollutant exposure on cognition. All models were adjusted for potential confounders: age (years) as a time-varying continuous covariate, smoking status (current, former and never smokers) and physical activity (sedentary, moderately active, very active) were included as time-varying categorical variables, whilst gender as recorded at baseline (categorical; females as the reference category) and the number of interviews provided (continuous) were also adjusted for. Wood et al. [23] also provide sensitivity analysis and discussion on the inclusion of a marker of socioeconomic status, although it was not adjusted for in the present study as it was only available for a subset of individuals and did not alter the results when adjusted for. All effect estimates were expressed per interquartile range (IQR) increase in exposure to provide results for a plausible increase in exposure, given that the mean levels and range of the two exposure metrics differed. London-dwelling ELSA respondents with complete covariate information were included in analyses (*n* = 768). All analyses were conducted in R version 4.2.1 [34].

## Results

A total of 768 London-dwelling ELSA participants were included in the present study, with a mean (±SD) age at the time of recruitment of 64.4 (±10.2) years and 57% of the sample being female (Table 1). The data provided in the present study covered a period of up to 15 years of follow-up with an average of 5.6 (±2.3) interviews undertaken per participant. Almost half of the sample population reported being moderately active at baseline and the majority of participants were either former (43%) or current (20.2%) smokers.

**Table 1:** Baseline descriptive statistics for demographic and interview data for ELSA respondents included in analyses of cognitive function.

	London-dwelling ELSA respondents
Number of participants	768
Number of interviews provided, mean ± SD	5.63 ± 2.26
Years of follow-up, mean ± SD	10.10 ± 4.57
Sex	
Women, *n* (%)	438 (57.0%)
Men, *n* (%)	330 (43.0%)
Age at recruitment (years), mean ± SD	64.40 ± 10.18
Baseline physical activity level	
Sedentary, *n* (%)	214 (27.9%)
Moderately active, *n* (%)	343 (44.6%)
Very active, *n* (%)	211 (27.5%)
Baseline smoking status	
Never smoked, *n* (%)	283 (36.8%)
Former smoker, *n* (%)	330 (43.0%)
Current smoker, *n* (%)	155 (20.2%)

An average of 11.98 and 11.12 LTDS respondents aged 50–64 and 65+ years old, respectively, provided time-activity data per postcode sector (Supplementary Material Table S5). The mean personal exposure factors assigned to ELSA participants for both age groups are provided in Table 2. Through indirect measures of time-activity patterns and mobility (based on age and residence within London), ELSA participants aged 50–64 years were estimated to be exposed to approximately 62%, 66% and 37% lower concentrations of NO_2_, PM_10_ and PM_2.5_, respectively, on average in comparison to exposure at the residential address when microenvironment exposures were estimated via the LHEM. Similar estimated reductions were observed for those aged 65 years and older.

**Table 2:** Summary statistics of baseline personal exposure factors derived from the LTDS and the LHEM, aggregated by age and residential postcode sector and assigned to London-dwelling ELSA participants; separated by age group.

	Min.	1st Qu.	Median	Mean ± SD	3rd Qu.	Max.
**Age group 50**–**64 (*n*** = **432)**						
*NO*_*2*_	0.258	0.365	0.384	0.382 ± 0.037	0.400	0.533
*PM*_*10*_	0.247	0.300	0.326	0.335 ± 0.051	0.357	0.515
*PM*_*2.5*_	0.511	0.611	0.622	0.625 ± 0.036	0.639	0.748
**Age Group 65**+ **(*n*** = **336)**						
*NO*_*2*_	0.242	0.344	0.359	0.359 ± 0.029	0.378	0.509
*PM*_*10*_	0.233	0.279	0.303	0.309 ± 0.040	0.328	0.447
*PM*_*2.5*_	0.512	0.598	0.607	0.608 ± 0.026	0.618	0.712

Mean baseline residence-based CMAQ-urban concentrations assigned to ELSA participants are provided in Table 3. Application of age group and postcode sector aggregated personal exposure factors to each ELSA participant’s residence-based estimate provided estimates of personalised exposure to ambient pollutants (LHEM-adjusted CMAQ-urban concentrations; Table 3). Average baseline concentrations were markedly reduced, by 63.4%, 67.8% and 38.8% for NO_2_, PM_10_ and PM_2.5_, on average respectively, when accounting for time-activity patterns in comparison to residence-based estimates alone. LHEM-adjusted estimates of personal exposure to ambient concentrations were highly correlated with modelled residence-based estimates for NO_2_ and PM_2.5_ (0.92 and 0.94, respectively), but the correlation was lower for PM_10_ (0.68; Table 3).

**Table 3:** Baseline CMAQ-urban and LHEM-adjusted CMAQ-urban pollutant concentrations (µg/m^3^) linked to ELSA respondents’ postcodes of residence, alongside overall IQRs per pollutant accounting for inter- and intra-respondent measurements and Spearman correlation coefficients between assigned residence-based CMAQ-urban concentrations and LHEM-adjusted estimates.

	Pollutant	Baseline mean ± SD [µg/m^3^]	IQR [µg/m^3^]	Spearman correlation with residential concentration
**CMAQ-urban residential concentration**	NO_2_	42.45 ± 14.69	11.10	
	PM_10_	21.21 ± 8.23	2.35	
	PM_2.5_	16.76 ± 5.88	2.50	
**LHEM-adjusted CMAQ-urban concentration**	NO_2_	15.54 ± 4.54	3.87	0.92
	PM_10_	6.83 ± 2.65	1.43	0.68
	PM_2.5_	10.26 ± 3.25	1.47	0.94

**Fig. 1:**
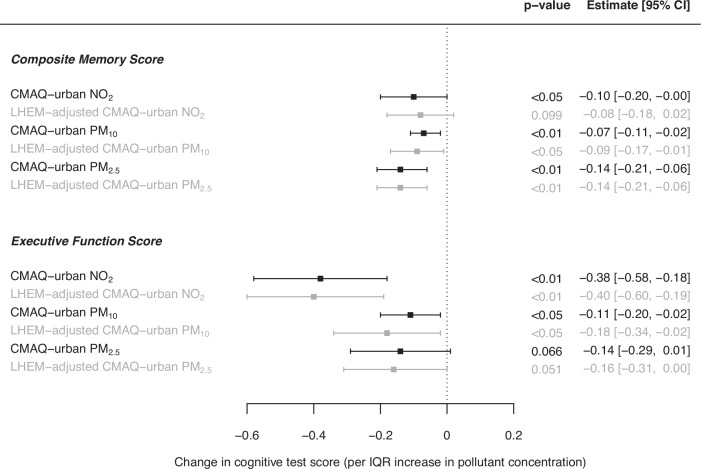
Associated effects of air pollution (residential and personalised estimates of exposure) on cognitive function in London-dwelling respondents of ELSA. Change in composite memory and executive function scores per IQR increase in residential NO_2_ (11.10 μg/m^3^), PM_10_ (2.35 μg/m^3^), PM_2.5_ (2.50 μg/m^3^), and personalised NO_2_ (3.87 µg/m^3^), PM_10_ (1.43 µg/m^3^) and PM_2.5_ (1.47 µg/m^3^) concentrations: single pollutant mixed-effects model effect estimates for 768 London-dwelling ELSA participants, adjusted for age, gender, number of interviews, smoking status and physical activity.

Baseline cognitive test performance in London-dwelling ELSA respondents is provided in Supplementary Material Table S8. Increased long-term exposures to residence-based CMAQ-urban modelled NO_2_, PM_10_ and PM_2.5_ were associated with decreased scores in both memory and executive function tests (Fig. 1). Interquartile range (IQR) increases in NO_2_ (11.10 μg/m^3^), PM_10_ (2.35 μg/m^3^) and PM_2.5_ (2.50 μg/m^3^) were associated with a decrease of −0.10 [95% CI: −0.20, 0.00], −0.07 [−0.11, −0.02] and −0.14 [−0.21, −0.06], respectively, in composite memory score. Similar decreases were observed for executive function scores of −0.38 [−0.58, −0.18], −0.11 [−0.20, −0.02] and −0.14 [−0.29, 0.01], respectively.

Associations with cognitive test performance when applying personalised exposure estimates were similar for composite memory scores, but a consistent pattern of slightly more adverse effects with executive function scores were observed. The largest difference was observed in the association between PM_10_ exposure and executive function score, in which the personalised exposure estimates were associated with a decrease of −0.18 [−0.34, −0.02] in executive function score (compared to −0.11 [−0.20, −0.02] when the residential estimate was used) per IQR increase (1.43 µg/m^3^).

## Discussion

The present study developed a framework through which personalised estimates of exposure to ambient air pollution concentrations, accounting for indirect information on time-activity and population mobility derived from a representative sample, could be applied to residence-based estimates traditionally assigned to individuals participating in an epidemiological cohort study. Application of this framework in a London-dwelling subset of a national English cohort provided a comparison in health effect estimates when assigning both exposure assessment methods to investigate the associations between ambient air pollution exposure and cognitive function. Adverse effects of exposure to NO_2_, PM_2.5_ and PM_10_ associated with cognitive function were found in a longitudinal cohort study with long follow-up and repeated measurements. Associations with cognitive test performance when applying personalised exposure estimates were similar for composite memory scores, but a consistent pattern of slightly more adverse effects with executive function scores were observed. To provide context, the decline in memory and executive function scores observed per IQR increase in long-term residential NO_2_ exposure was found to be equivalent to that of ageing by approximately 1.5 and 4 years, respectively, in the ELSA cohort [23].

Personalised concentration estimates were found to be markedly lower than residence-based outdoor concentration estimates on average for all pollutants investigated. This is likely due to the fact that Londoners spend up to 95% of their time indoors [10] where exposure to ambient pollutants is lower depending on factors such as the indoor/outdoor ratio of concentrations and infiltration efficiency [13]. A recently published review of studies aiming to personalise estimates of exposure to ambient air pollution also reports that individuals spend the majority of their time indoors at home (60–70%), as well as a large proportion of their time in other indoor environments, although these proportions differ between population groups and individuals [35]. The LHEM has not been validated against personal measurements given the difficulty in designing a personal monitoring campaign of the required size to perform such validation. The present study is unaware of any such study to date. However, CMAQ-urban has been validated against measured concentrations [6] (Supplementary Material Fig. S1 and Table S1). Additionally, the LHEM model utilises several microenvironment models to estimate exposure to ambient sources when indoors in specific building types, as well as when inside different vehicles. Infiltration rates of ambient pollutants into indoor environments differ by both building and vehicle type, as well as by pollutant, which is accounted for by an indoor/outdoor building infiltration model [33] that accounts for 15 Greater London-dwelling archetypes (covering 76% of the known London-dwelling stock) and several independent mass balance equations for each transportation mode. Measurement campaign data conducted across the London Underground network were also included in LHEM modelling of microenvironment concentrations [15].

Previous work investigating personalised exposure in comparison to outdoor residence-based estimates has often not found such marked differences in estimated concentrations between exposure assessment methods [17, 18, 35]. However, infiltration of ambient concentrations to indoor environments is often not taken into account and methodologies employed in apportioning microenvironment exposure (as well as the microenvironments accounted for) currently differ greatly between such studies. In fact, assessing personalised exposure, even if making no significant difference in the effect estimates, reflects a more accurate exposure, in terms of magnitude, to real personal exposure.

Despite the observed reductions in concentrations of personalised exposures to each pollutant in comparison to outdoor residence-based estimates in the present study, correlations between the two were high for NO_2_ (0.92) and PM_2.5_ (0.94), but somewhat lower for PM_10_ (0.68). Previous studies have reported similarly high correlations for NO_2_ in adult populations [17, 18], as well as for PM_2.5_ in children [19, 20]. No previous work identified by the present study has reported on the correlation between residence-based and personalised estimates of PM_10_.

High correlations between the two exposure assessment methods likely explain the similarities observed in effect estimates and such findings may suggest that the inclusion of time-activity information into personalised exposure estimates may not improve epidemiological analyses assigning residence-based estimates only. Furthermore, if the difference between personalised and residence-based estimates is relatively uniform across individuals and does not greatly affect the rank order in exposure per pollutant, then a difference in health effect estimates between the two methods would not necessarily be expected. It should also be noted that the assignment of ambient concentrations in epidemiological studies provides surrogates of exposure and may not necessarily reflect true personal exposure, which is likely lower, for many of the reasons described in the present study regarding time spent in various microenvironments. The personalised concentrations assigned in the present study (which are lower than modelled ambient concentrations) cannot be interpreted as having a “smaller” effect on cognition, as they are a reflection of the surrogate measures, hence effect estimates are presented by IQR and not by a specific change in concentration.

Previous work has generally reported similar findings to that of the present study, with the application of personalised exposure providing little difference in health effect estimates in comparison to that of residence-based estimates for mortality [21, 22] and markers of inflammation [36] in adult populations, as well as lung function [20] and asthma [37] in children. Letellier et al. [38] did find associations in cardio-metabolic markers when assigning personalised exposure in comparison to no associations observed for ambient residence-based estimates. However, the study assigned residence-based estimates using an average value within a 1600 m buffer around the residence, which may not capture the necessary fine-scale variations in outdoor concentrations between participants required for accurate health effect estimation when using residence-based estimates of exposure. The present study utilised fine-scale spatial modelling of ambient concentrations at a 20 × 20 m scale.

Adaptation of the LTDS as an external data source of population time-activity and the microenvironment modelling capabilities of the LHEM presented here provide a number of advantages over previous studies aiming to personalise assigned exposures to ambient air pollution using travel surveys. Shekarizzfard et al. [39] utilised travel survey data encompassing 15,572 trips (from 5945 individuals) in Montreal, Canada, for the year 2008 in order to model exposure trajectories for vehicle drivers/passengers, public transport users and active commuters using an integrated transportation and emissions model linked to a dispersion model. Comparison between daily average outdoor residence-based exposure to NO_2_ and time-weighted averages including time-activity patterns found 89.6% of individuals to be assigned lower concentrations when using the outdoor residential estimate only. Similarly, Shekarizzfard et al. [40] calculated time-weighted average exposure to black carbon (BC) and ultrafine particles (UFP) using 2011 travel survey data for 1,179,489 trips (341,274 individuals) in Toronto, Canada, finding the median mobility-based exposure to be 11.6% and 63.2% higher than outdoor residence-based exposure for UFP and BC, respectively. In both cases, exposure to ambient concentrations when indoors or in-vehicle was not taken into account, but ambient concentrations assigned at point-time locations without factoring in infiltration to indoor spaces were applied.

As previously described, the LHEM provides a suite of modelling procedures to estimate the infiltration of ambient air pollution into indoor and in-vehicle microenvironments. This provides a further advantage over previous studies that estimated ambient concentrations at several locations such as home/work/school and assigned time-weighted averages [5, 22, 39], which do not account for actual infiltration of ambient pollutants to indoor/in-vehicle environments. Lane et al. [36] did apportion time in separate microenvironments (inside/outside the home, at work, on the highway or other) to investigate the difference in exposure to UFP when accounting for time-activity in 140 residents of Boston, USA, with a mean age (59.1 years) comparable to that of the present study. Lower exposure to UFP was estimated for those spending a greater amount of time away from the home, however, the calculation of personalised exposure did differ from that of the present study. Residence-based estimates (based on proximity to a major highway) were assigned for workers assumed to be highly exposed to traffic-related air pollution (TRAP) when at work, vehicle type information was unknown and 100% infiltration was assigned when in-vehicle, infiltration to the home was inferred from air conditioning usage and urban background average residential concentrations were assigned to other microenvironments. Beckx et al. [41] utilised a model estimating time-activity patterns based on survey data (~10,000 person-day activity-diaries collected between 1997 and 2001), including the estimation of transportation mode for trips, which was extrapolated to a synthetic population with inferred residential information. Residential modelled concentrations were then compared to personalised exposures (constructed from hourly dynamic exposures accounting for time-activity) for the city of Utrecht in the Netherlands. The results in Beckx et al. [41] are reported in terms of person-hours spent above concentration thresholds for PM_10_ and PM_2.5_ for April 2005, with marked increases in time spent above such thresholds observed for personalised exposure in comparison to residence-based estimates.

The LTDS survey provides time-activity information for a large number of participants across multiple years and is a continually ongoing study [10]. The ability of the LHEM to model exposure to ambient air pollutants across a range of microenvironments allows for an almost complete modelled picture of exposure to ambient concentrations and does not require time-weighting of ambient concentrations based solely on point-time location. The capabilities of the LHEM to account for infiltration into indoor or in-vehicle microenvironments provides a plausible explanation for the fact that the present study observed a marked reduction in assigned personalised exposures in comparison to outdoor residence-based estimates; a finding that has not been consistently reported in the literature [17, 18, 39].

Additionally, the epidemiological analysis of cognitive function in the present study utilised a cohort providing up to 15 years of follow-up and 5.6 repeated measures on average. Epidemiological studies of cognitive function thus far generally rely on cross-sectional study designs or longitudinal studies with fewer repeated measures and both of these issues have been discussed in the literature as potential weaknesses that must be addressed in future work [4].

The present study utilised external travel survey data to estimate personalised exposure to ambient air pollutants [10] and applied the estimates to an epidemiological analysis within a cohort. Indirectly estimating personal exposure is a crucial issue as the ability to measure it is often not viable, given the cost involved and the inconvenience it would place on individuals over a long enough period of time. Other methodologies have been previously employed to indirectly estimate time-activity patterns and personalise exposure to ambient air pollution, such as agent-based modelling [19, 42], tracking studies [43] and studies collecting information to estimate time spent between the home and work/school address [22]. However, the use of the LTDS in the present study allowed a breakdown of each individual’s survey day at minute-by-minute resolution, given the detailed collection of trip origin/destination, home/work address and transportation mode, as well as hourly modelling of residential concentrations at a fine spatial scale.

Application of the LTDS and LHEM data to personalise outdoor residence-based estimates does however include several limitations. Principally, the application of LTDS survey data and the LHEM in the present study utilised just age and area of residence to personalise exposures, assuming that these two factors have an impact on the difference between residential concentrations and those adjusted for time-activity patterns. This is likely not the case and future work will explore the use of other information available in the LTDS (such as gender and markers of socioeconomic status) to aggregate exposure factors and investigate the impact on the adjustment of residence-based exposure estimates. At present, the LHEM assigns hourly ambient CMAQ-urban estimated residential concentrations for each LTDS participant for the year 2011, whilst the LTDS data set used spanned 2005–2010. This assumes that the spatial variation of outdoor residential concentrations did not markedly change between year of interview and 2011, as well as expecting that representative time-activity and population mobility information derived for these years applies to the years of ELSA follow-up that this period did not cover (2002–2004 and 2011–2017). One further limitation imposed by the necessity for ELSA participant data to remain anonymised was the categorisation of residence-based estimates based on the distribution of assigned postcode estimates. This process may have affected the adjustment of residential modelled estimates by diluting the between-person variability in exposure to some extent (discussed further in Wood et al. [23]).

The LHEM models point-time exposure to ambient air pollutants at minute-by-minute resolution for LTDS individuals when outdoors, indoors and in several transport microenvironments. It does not model exposure to indoor-generated air pollution. Investigation into indoor-generated air pollution in London homes estimated indoor sources to increase indoor concentrations of NO_2_, PM_10_ and PM_2.5_ by 26–37% [44]. The aim of the present study was to assess the potential for utilising the LTDS and LHEM to indirectly adjust modelled ambient residence-based concentrations which are often assigned in epidemiological studies, allowing for comparisons between health effect estimates using a more traditional approach and one aimed at personalising ambient exposure estimation through the inclusion of time-activity and population mobility information. Adaptation of the LHEM to incorporate indoor-generated air pollution in future work would give a more complete picture of total personal exposure but this was not the aim of the present study.

The incorporation of time-activity information to personalise exposure assessment in epidemiological study generally relies on the inclusion of indirect information and this is unlikely to change in the near future. Direct measurement of personal exposure is currently difficult to achieve for the purposes of viable epidemiological analyses. The development of methodologies to accomplish the accurate personalisation of estimates will depend on the availability of data from sources such as detailed time-activity surveys and the application of microenvironment modelling techniques. The present study provides a novel framework and case study comparing the assignment of residence-based and personalised estimates in an epidemiological analysis of cognitive function, finding little difference in health effect estimates. Future work will aim to develop the presented framework and assess the calculation of personal exposure in London-dwelling individuals by factors other than age and area of residence, as well as applying the framework to other health outcomes.

## Supplementary information

Supplementary material

## Data Availability

ELSA data are available to researchers via the UK Data Service: https://www.elsa-project.ac.uk/accessing-elsa-data. LTDS data are managed by Transport for London. Additional data are available from the corresponding author upon reasonable request.

## References

[1] Chen J, Hoek G. Long-term exposure to PM and all-cause and cause-specific mortality: a systematic review and meta-analysis. Environ Int. 2020;143:105974. 32703584 10.1016/j.envint.2020.105974

[2] de Bont J, Jaganathan S, Dahlquist M, Persson Å, Stafoggia M, Ljungman P. Ambient air pollution and cardiovascular diseases: An umbrella review of systematic reviews and meta‐analyses. J Intern Med. 2022;291:779–800. 35138681 10.1111/joim.13467 PMC9310863

[3] Delgado-Saborit JM, Guercio V, Gowers AM, Shaddick G, Fox NC, Love S. A critical review of the epidemiological evidence of effects of air pollution on dementia, cognitive function and cognitive decline in adult population. Sci Total Environ. 2021;757:143734. 33340865 10.1016/j.scitotenv.2020.143734

[4] Weuve J, Bennett EE, Ranker L, Gianattasio KZ, Pedde M, Adar SD, et al. Exposure to air pollution in relation to risk of dementia and related outcomes: an updated systematic review of the epidemiological literature. Environ Health Perspect. 2021;129:096001. 34558969 10.1289/EHP8716 PMC8462495

[5] Reis S, Liška T, Vieno M, Carnell EJ, Beck R, Clemens T, et al. The influence of residential and workday population mobility on exposure to air pollution in the UK. Environ Int. 2018;121:803–13. 30340197 10.1016/j.envint.2018.10.005

[6] Dimakopoulou K, Samoli E, Analitis A, Schwartz J, Beevers S, Kitwiroon N, et al. Development and evaluation of spatio-temporal air pollution exposure models and their combinations in the greater London Area, UK. Int J Environ Res Public Health. 2022;19:5401. 35564796 10.3390/ijerph19095401 PMC9103954

[7] Chatzidiakou L, Krause A, Kellaway M, Han Y, Li Y, Martin E, et al. Automated classification of time-activity-location patterns for improved estimation of personal exposure to air pollution. Environ Health. 2022;21:125. 36482402 10.1186/s12940-022-00939-8 PMC9733291

[8] Lu Y. Beyond air pollution at home: assessment of personal exposure to PM2. 5 using activity-based travel demand model and low-cost air sensor network data. Environ Res. 2021;201:111549. 34153337 10.1016/j.envres.2021.111549

[9] Domínguez A, Koch S, Marquez S, de Castro M, Urquiza J, Evandt J, et al. Childhood exposure to outdoor air pollution in different microenvironments and cognitive and fine motor function in children from six European cohorts. Environ Res. 2024;247:118174. 38244968 10.1016/j.envres.2024.118174

[10] Smith JD, Mitsakou C, Kitwiroon N, Barratt BM, Walton HA, Taylor JG, et al. London hybrid exposure model: improving human exposure estimates to no2 and pm2. 5 in an urban setting. Environ Sci Technol. 2016;50:11760–8. 27706935 10.1021/acs.est.6b01817

[11] Korhonen A, Relvas H, Miranda AI, Ferreira J, Lopes D, Rafael S, et al. Analysis of spatial factors, time-activity and infiltration on outdoor generated PM2. 5 exposures of school children in five European cities. Sci Total Environ. 2021;785:147111. 33940420 10.1016/j.scitotenv.2021.147111

[12] Ferguson L, Taylor J, Zhou K, Shrubsole C, Symonds P, Davies M, et al. Systemic inequalities in indoor air pollution exposure in London, UK. Build Cities. 2021;2:425. 34124667 10.5334/bc.100 PMC7610964

[13] Chen C, Zhao B. Review of relationship between indoor and outdoor particles: I/O ratio, infiltration factor and penetration factor. Atmos Environ. 2011;45:275–88.

[14] De Nazelle A, Bode O, Orjuela JP. Comparison of air pollution exposures in active vs. passive travel modes in European cities: a quantitative review. Environ Int. 2017;99:151–60. 28043651 10.1016/j.envint.2016.12.023

[15] Smith JD, Barratt BM, Fuller GW, Kelly FJ, Loxham M, Nicolosi E, et al. PM2. 5 on the London Underground. Environ Int. 2020;134:105188. 31787325 10.1016/j.envint.2019.105188 PMC6902242

[16] Mak J, Feary J, Amaral AF, Marczylo E, Cullinan P, Green DC. Occupational exposure to particulate matter and staff sickness absence on the London underground. Environ Int. 2024;185:108529. 38484612 10.1016/j.envint.2024.108529

[17] Ragettli MS, Phuleria HC, Tsai MY, Schindler C, De Nazelle A, Ducret-Stich RE, et al. The relevance of commuter and work/school exposure in an epidemiological study on traffic-related air pollution. J Expo Sci Environ Epidemiol. 2015;25:474–81. 25492241 10.1038/jes.2014.83

[18] Blanchard O, Deguen S, Kihal-Talantikite W, François R, Zmirou-Navier D. Does residential mobility during pregnancy induce exposure misclassification for air pollution? Environ Health. 2018;17:1–6. 30340597 10.1186/s12940-018-0416-8 PMC6194718

[19] Ntarladima AM, Vaartjes I, Grobbee DE, Dijst M, Schmitz O, Uiterwaal C, et al. Relations between air pollution and vascular development in 5-year old children: a cross-sectional study in the Netherlands. Environ Health. 2019;18:1–2. 31096974 10.1186/s12940-019-0487-1 PMC6524285

[20] Ntarladima AM, Karssenberg D, Vaartjes I, Grobbee DE, Schmitz O, Lu M, et al. A comparison of associations with childhood lung function between air pollution exposure assessment methods with and without accounting for time-activity patterns. Environ Res. 2021;202:111710. 34280420 10.1016/j.envres.2021.111710

[21] Puett RC, Hart JE, Suh H, Mittleman M, Laden F. Particulate matter exposures, mortality, and cardiovascular disease in the health professionals follow-up study. Environ Health Perspect. 2011;119:1130–5. 21454146 10.1289/ehp.1002921 PMC3237347

[22] Christidis T, Pinault LL, Crouse DL, Tjepkema M. The influence of outdoor PM2. 5 concentration at workplace on nonaccidental mortality estimates in a Canadian census-based cohort. Environ Epidemiol. 2021;5:e180. 34909560 10.1097/EE9.0000000000000180 PMC8663884

[23] Wood D, Evangelopoulos D, Beevers S, Kitwiroon N, Demakakos P, Katsouyanni K. Exposure to ambient air pollution and cognitive function: an analysis of the English Longitudinal Study of Ageing cohort. Environ Health. 2024;23:35. 38575976 10.1186/s12940-024-01075-1 PMC10996194

[24] Marmot M, Banks J, Blundell R, Lessof C, Nazroo J. Health, wealth and lifestyles of the older population in England. London: Institute of Fiscal Studies; 2003.

[25] Steptoe A, Breeze E, Banks J, Nazroo J. Cohort profile: the English longitudinal study of ageing. Int J Epidemiol. 2013;42:1640–8. 23143611 10.1093/ije/dys168 PMC3900867

[26] Maharani A, Tampubolon G. National economic development status may affect the association between central adiposity and cognition in older adults. PLoS ONE. 2016;11:e0148406. 26863443 10.1371/journal.pone.0148406 PMC4749166

[27] Olaya B, Bobak M, Haro JM, Demakakos P. Trajectories of verbal episodic memory in middle‐aged and older adults: evidence from the English Longitudinal Study of Ageing. J Am Geriatr Soc. 2017;65:1274–81. 28263362 10.1111/jgs.14789

[28] Yin J, Lassale C, Steptoe A, Cadar D. Exploring the bidirectional associations between loneliness and cognitive functioning over 10 years: the English longitudinal study of ageing. Int J Epidemiol. 2019;48:1937–48. 31056641 10.1093/ije/dyz085 PMC6929532

[29] Ray J, Popli G, Fell G. Association of cognition and age-related hearing impairment in the English Longitudinal Study of Ageing. JAMA Otolaryngol Head Neck Surg. 2018;144:876–82. 30193368 10.1001/jamaoto.2018.1656 PMC6233824

[30] Zheng F, Yan L, Yang Z, Zhong B, Xie W. HbA1c, diabetes and cognitive decline: the English Longitudinal Study of Ageing. Diabetologia. 2018;61:839–48. 29368156 10.1007/s00125-017-4541-7 PMC6448974

[31] Beevers SD, Kitwiroon N, Williams ML, Carslaw DC. One way coupling of CMAQ and a road source dispersion model for fine scale air pollution predictions. Atmos Environ. 2012;59:47–58. 10.1016/j.atmosenv.2012.05.034 PMC3587455 23471172

[32] Fairnie GA, Wilby DJ, Saunders LE. Active travel in London: the role of travel survey data in describing population physical activity. J Transp Health. 2016;3:161–72.

[33] Taylor J, Shrubsole C, Davies M, Biddulph P, Das P, Hamilton I, et al. The modifying effect of the building envelope on population exposure to PM 2.5 from outdoor sources. Indoor Air. 2014;24:639–51. 24713025 10.1111/ina.12116 PMC4278446

[34] R Core Team. R: A language and environment for statistical computing. Vienna, Austria: R Foundation for Statistical Computing; 2022.

[35] Hoek G, Vienneau D, de Hoogh K. Does residential address-based exposure assessment for outdoor air pollution lead to bias in epidemiological studies? Environ Health. 2024;23:75. 39289774 10.1186/s12940-024-01111-0 PMC11406750

[36] Lane KJ, Levy JI, Scammell MK, Patton AP, Durant JL, Mwamburi M, et al. Effect of time-activity adjustment on exposure assessment for traffic-related ultrafine particles. J Expo Sci Environ Epidemiol. 2015;25:506–16. 25827314 10.1038/jes.2015.11 PMC4542140

[37] McConnell R, Islam T, Shankardass K, Jerrett M, Lurmann F, Gilliland F, et al. Childhood incident asthma and traffic-related air pollution at home and school. Environ Health Perspect. 2010;118:1021–6. 20371422 10.1289/ehp.0901232 PMC2920902

[38] Letellier N, Zamora S, Spoon C, Yang JA, Mortamais M, Escobar GC, et al. Air pollution and metabolic disorders: dynamic versus static measures of exposure among Hispanics/Latinos and non-Hispanics. Environ Res. 2022;209:112846. 35120894 10.1016/j.envres.2022.112846 PMC8976727

[39] Shekarrizfard M, Faghih-Imani A, Hatzopoulou M. An examination of population exposure to traffic related air pollution: Comparing spatially and temporally resolved estimates against long-term average exposures at the home location. Environ Res. 2016;147:435–44. 26970897 10.1016/j.envres.2016.02.039

[40] Shekarrizfard M, Minet L, Miller E, Yusuf B, Weichenthal S, Hatzopoulou M. Influence of travel behaviour and daily mobility on exposure to traffic-related air pollution. Environ Res. 2020;184:109326. 32155490 10.1016/j.envres.2020.109326

[41] Beckx C, Panis LI, Arentze T, Janssens D, Torfs R, Broekx S, et al. A dynamic activity-based population modelling approach to evaluate exposure to air pollution: methods and application to a Dutch urban area. Environ Impact Assess Rev. 2009;29:179–85.

[42] Lu M, Schmitz O, Vaartjes I, Karssenberg D. Activity-based air pollution exposure assessment: differences between homemakers and cycling commuters. Health Place. 2019;60:102233. 31675651 10.1016/j.healthplace.2019.102233

[43] Marquet O, Tello-Barsocchini J, Couto-Trigo D, Gómez-Varo I, Maciejewska M. Comparison of static and dynamic exposures to air pollution, noise, and greenness among seniors living in compact-city environments. Int J Health Geogr. 2023;22:3. 36709304 10.1186/s12942-023-00325-8 PMC9884423

[44] Vu TV, Stewart GB, Kitwiroon N, Lim S, Barratt B, Kelly FJ, et al. Assessing the contributions of outdoor and indoor sources to air quality in London homes of the SCAMP cohort. Build Environ. 2022;222:109359.

