# Supplementary material for: Personalised estimation of exposure to ambient air pollution and application in a longitudinal cohort analysis of cognitive function in London-dwelling older adults
Source: J Expo Sci Environ Epidemiol. 2025 Jan 14;36(1):33–40. doi: 10.1038/s41370-025-00745-7 (PMC12795752; doi:10.1038/s41370-025-00745-7)
Supplement: Supplementary file 1 — Supplementary material [file 41370_2025_745_MOESM1_ESM.docx]

**LHEM methods paper supplementary material**


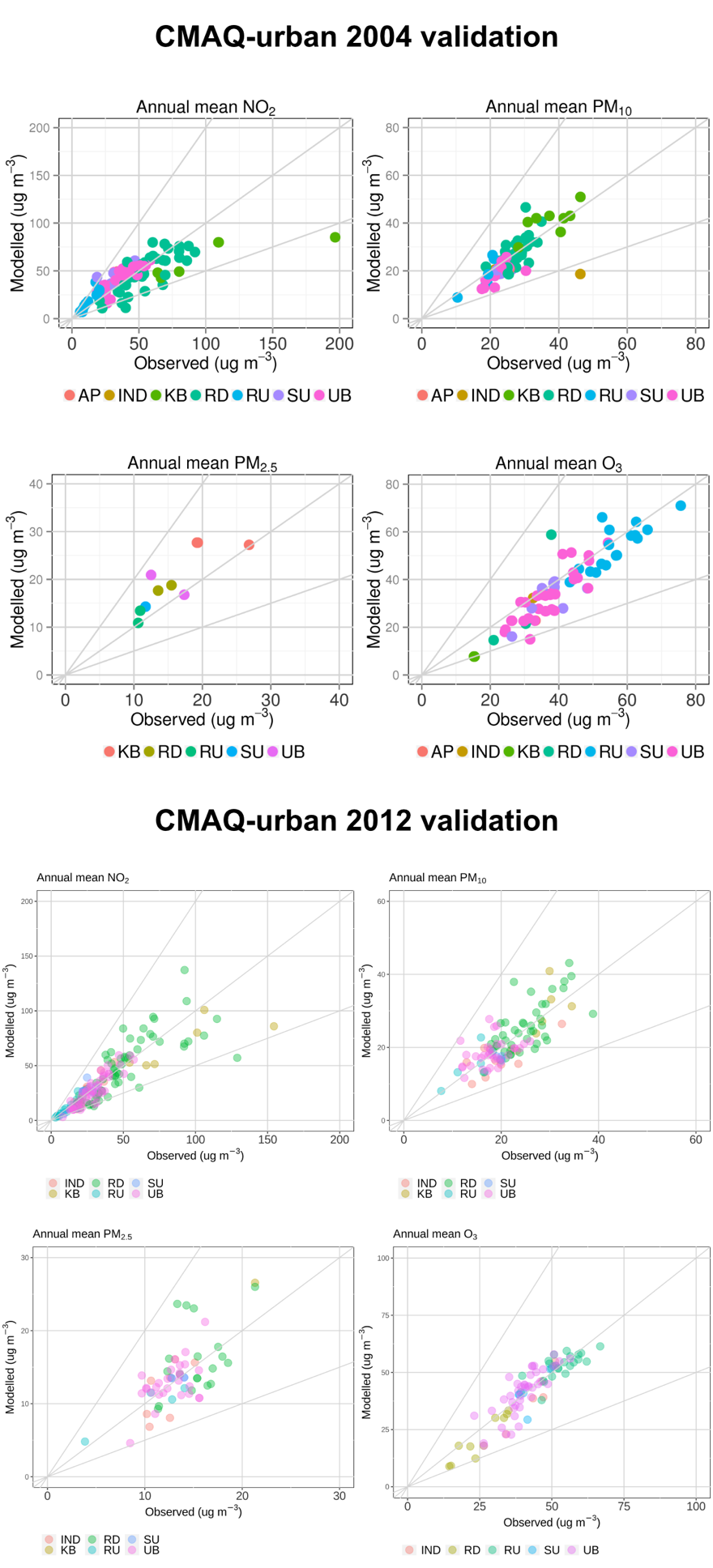


**Figure S1.** CMAQ-urban 2004 and 2012 model validation. Annual average observed (monitor concentrations) plotted against annual average modelled concentrations at the 20x20m grid cell in which the monitor is located. Monitor site type denoted by colour. **AP**: airport; **IND**: industrial; **KB**: kerbside; **RD**: roadside; **RU**: rural; **SU**: suburban; **UB**: urban background.

**Table S1.** CMAQ-urban 2004 and 2012 model performance and validation in comparison to measured concentrations of NO_2_, PM_10_ and PM_2.5_.

|  | **2004** | | |  | **2012** |  |
| --- | --- | --- | --- | --- | --- | --- |
| **Pollutant** | **N Monitor Sites** | **RMSE** | **r** | **N Monitor Sites** | **RMSE** | **r** |
| **NO_2_** | 122 | 15.32 | 0.78 | 155 | 13.32 | 0.86 |
| **PM_10_** | 88 | 5.13 | 0.78 | 102 | 4.50 | 0.78 |
| **PM_2.5_** | 10 | 5.04 | 0.82 | 63 | 3.17 | 0.67 |

**Table S2**. Annual average residence-based concentrations of NO_2_, PM_10_ and PM_2.5_ for 2004 estimated by CMAQ-urban categorised into England-wide deciles per pollutant (deciles 9 and 10 expanded) assigned to London-dwelling ELSA participants (n = 768) at baseline.

|  | **Categorical exposure variable** | **Range**  **[ug/m^3^]** | **Mid-range concentration [ug/m^3^]** | **ELSA participants at baseline** |
| --- | --- | --- | --- | --- |
| **NO_2_** |  |  |  |  |
|  | **5** | 22.4-25.5 | 23.95 | 8 |
|  | **6** | 25.5-28.5 | 27 | 32 |
|  | **7** | 28.5-32.5 | 30.5 | 50 |
|  | **8** | 32.5-37.5 | 35 | 274 |
|  | **9.1** | 37.5-40.5 | 39 | 104 |
|  | **9.2** | 40.5-44.8 | 42.65 | 116 |
|  | **10.1** | 44.8-50.3 | 47.55 | 113 |
|  | **10.2** | 50.3-119.1 | 84.7 | 72 |
| **PM_10_** |  |  |  |  |
|  | **4** | 16.1-16.7 | 16.4 | 13 |
|  | **5** | 16.7-17.3 | 17 | 22 |
|  | **6** | 17.3-17.9 | 17.6 | 82 |
|  | **7** | 17.9-18.7 | 18.3 | 170 |
|  | **8** | 18.7-19.8 | 19.25 | 188 |
|  | **9.1** | 19.8-20.9 | 20.35 | 110 |
|  | **9.2** | 20.9-22.4 | 21.65 | 98 |
|  | **10.1** | 22.4-24.9 | 23.65 | 54 |
|  | **10.2** | 24.9-94.7 | 59.8 | 32 |
| **PM_2.5_** |  |  |  |  |
|  | **5** | 11.9-12.3 | 12.1 | 7 |
|  | **6** | 12.3-12.7 | 12.5 | 13 |
|  | **7** | 12.7-13.2 | 12.95 | 37 |
|  | **8** | 13.2-14 | 13.6 | 131 |
|  | **9.1** | 14-14.5 | 14.25 | 173 |
|  | **9.2** | 14.5-15.2 | 14.85 | 117 |
|  | **10.1** | 15.2-17 | 16.1 | 201 |
|  | **10.2** | 17-48.3 | 32.65 | 90 |

**Table S3**. Microenvironment classification and the LHEM components from which residence-based CMAQ-urban estimates were derived.


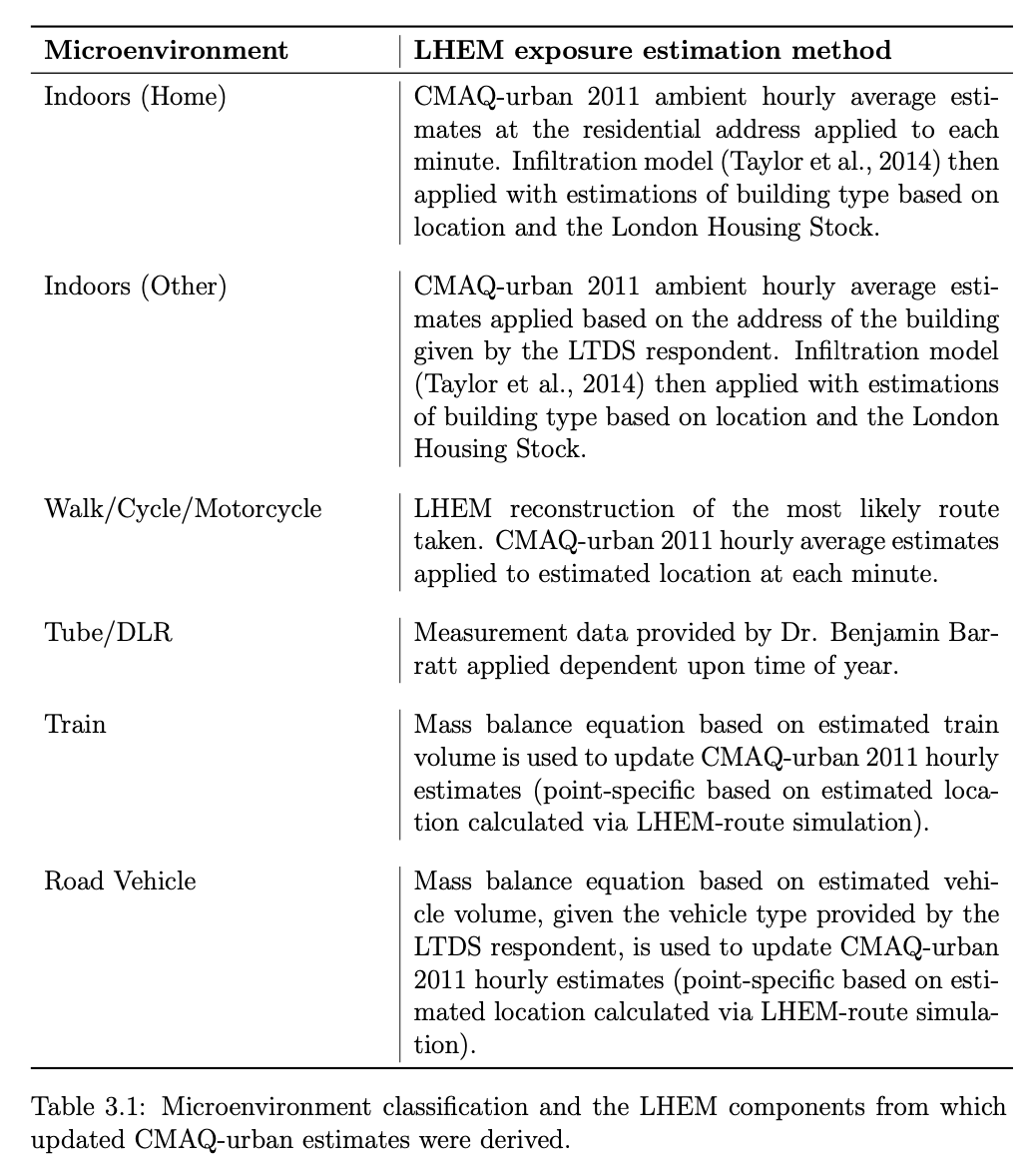


**Figure S2**. Age distribution of LTDS respondents aged 50 years and older (n = 19,349) and the number of respondents categorised into each age group.


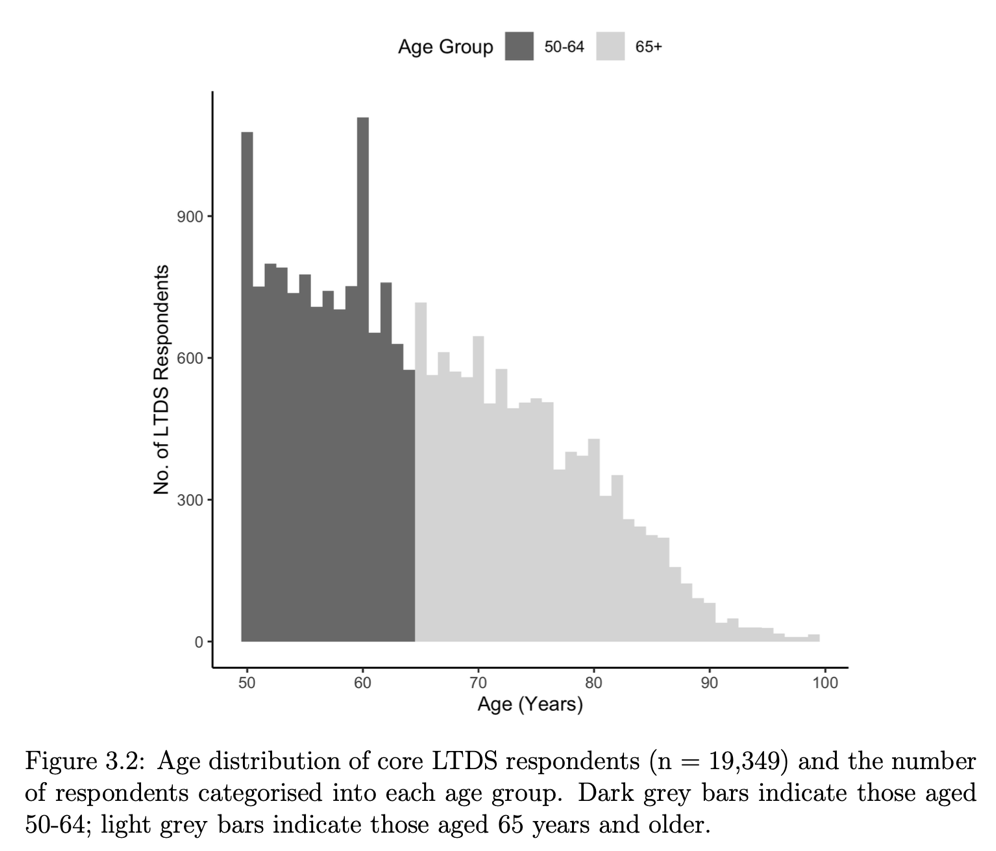


**Table S4**. Descriptive statistics of postcodes by London postcode sector.


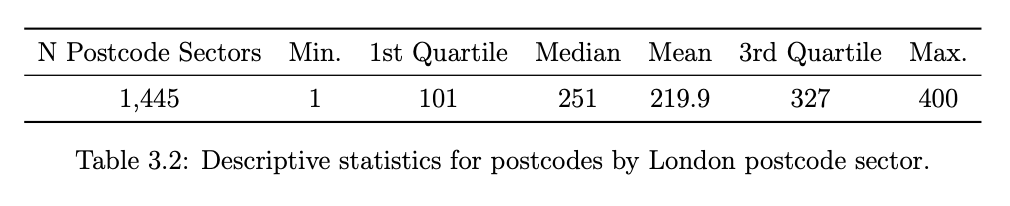


**Table S5**. Mean number of LTDS respondent providing time-activity information per age group, per London postcode sector.

**
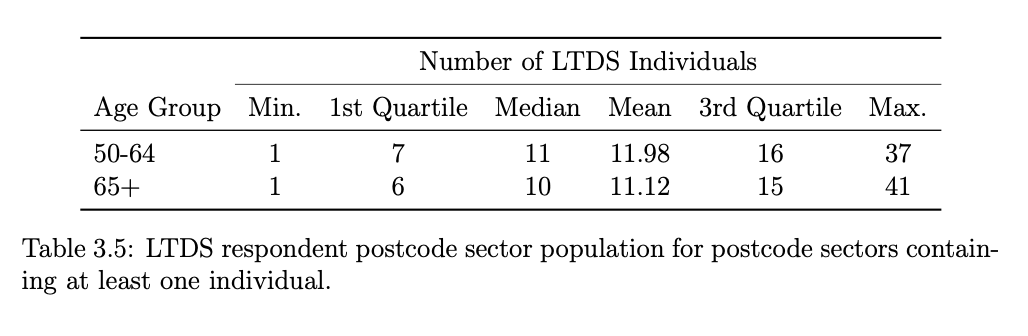
**

**Table S6**. Overall mean time spent in each microenvironment by LTDS respondents per age group.

**
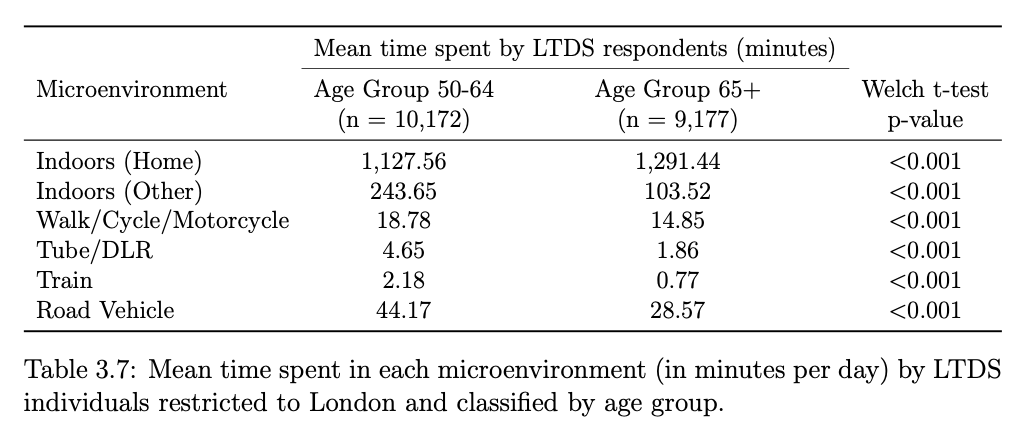
**

**Table S7**. Mean personal exposure factors calculated for LTDS respondents per age group, per pollutant.


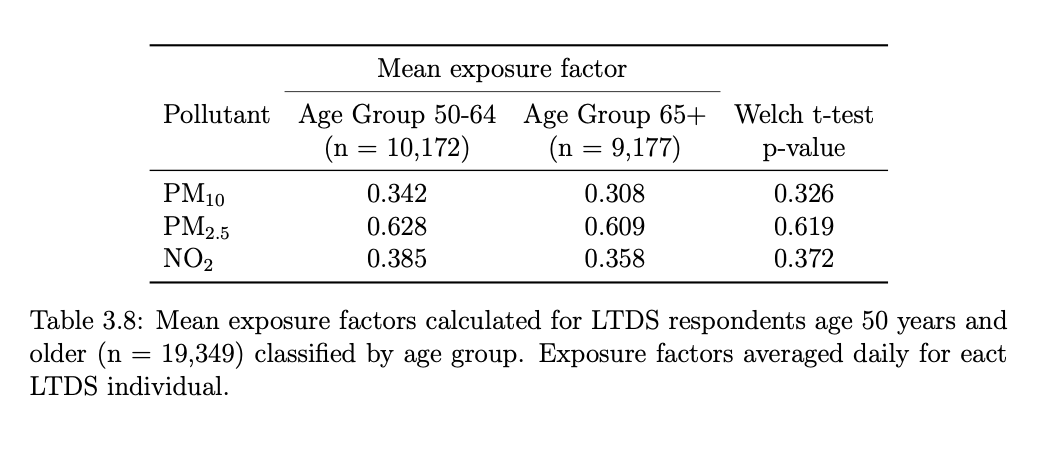


**Table S8.** Descriptive statistics of baseline cognitive test scores provided by ELSA respondents included in analyses of cognitive function across London (n = 768), separated by baseline age group.

|  |  |  |  |  |  |  |  |
| --- | --- | --- | --- | --- | --- | --- | --- |
| Baseline age (years) | n | Min. | 1^st^ Quartile | Mean ±SD | Median | 3^rd^ Quartile | Max. |
| Composite Memory Score (0 – 20) |  |  |  |  |  |  |  |
| 50-59 | 308 | 1 | 9 | 10.6 ±3.5 | 11 | 13 | 20 |
| 60-69 | 232 | 0 | 8 | 9.8 ±3.1 | 10 | 12 | 16 |
| 70-79 | 150 | 0 | 6 | 8.6 ±3.3 | 9 | 11 | 16 |
| 80-89 | 71 | 0 | 5 | 7.0 ±3.1 | 7 | 9 | 14 |
| 90+ | 7 | 4 | 6 | 7.1 ±2.0 | 8 | 8 | 10 |
|  |  |  |  |  |  |  |  |
| Executive Function Score |  |  |  |  |  |  |  |
| 50-59 | 308 | 0 | 17 | 20.9 ±7.0 | 21 | 25 | 50 |
| 60-69 | 232 | 5 | 15 | 19.0 ±6.4 | 18 | 23 | 40 |
| 70-79 | 150 | 1 | 13 | 17.5 ±5.8 | 17 | 21 | 35 |
| 80-89 | 71 | 1 | 11 | 15.6 ±6.1 | 15 | 20 | 37 |
| 90+ | 7 | 9 | 11 | 14.4 ±4.9 | 14 | 16 | 24 |
|  |  |  |  |  |  |  |  |
